# Moral Judgments Across the Economic Divide: The Effect of Perceived Economic Inequality and Status in Judgment of Transgressions, Justification, and Dehumanization

**DOI:** 10.3390/bs15101317

**Published:** 2025-09-26

**Authors:** Alba Álamo-Hernández, Mario Sainz, Verónica Betancor, Armando Rodríguez-Pérez

**Affiliations:** 1 Department of Cognitive, Social and Organizational Psychology, Faculty of Psychology, University of La Laguna, 38200 San Cristóbal de La Laguna, Spain; verbetan@ull.edu.es (V.B.); arguez@ull.edu.es (A.R.-P.); 2 Department of Social Psychology, Faculty of Psychology, University of Granada, 18011 Granada, Spain; mariosainz@ugr.es

**Keywords:** moral judgments, moral justification, dehumanization, economic inequality, socioeconomic status

## Abstract

Moral judgments are influenced by the context in which a transgression occurs and the characteristics of the transgressor. This study examined how economic inequality and the socioeconomic status (SES) of the transgressor affect the Judgement of the transgression, its Justification, and the Dehumanization of the transgressor. In addition, we investigated the mechanisms that mediate these effects, such as anomie, a sense of control, and sympathy. Through two experimental studies (Study 1: N = 289; Study 2: N = 401), we found that both perceived economic inequality and SES (low vs. high) influence moral judgments. Specifically, high-status transgressors are judged more harshly, their actions are less justified, and they are more dehumanized. We also determined that higher economic inequality increases moral Justifications through perceptions of greater anomie, whilst reduced sympathy toward high-status transgressors explains their harsher judgments and greater Dehumanization. This research helps our understanding of how inequality and SES shape moral judgments and highlights the importance of contextualizing them.

## 1. Introduction

To regulate their social lives, individuals rely on moral judgments to evaluate behaviors, distinguishing which actions are acceptable and which deserve condemnation (Malle, 2021). Those behaviors perceived to be morally appropriate and beneficial are typically judged positively, whilst those considered to be undesired or harmful elicit moral disapproval and negative judgments. As a result, immoral behaviors often provoke condemnation, punishment, and even the dehumanization of those committing them (Bastian et al., 2013; Khamitov et al., 2016). Nonetheless, not all transgressions result in social rejection or punishment. For instance, despite the legal accusations in the widely publicized tax fraud case involving the singer Shakira, she was not imprisoned, and she maintained a largely favorable public image among her followers. This example underscores the notion that moral judgments are not made in isolation but are influenced by broader social contexts and the transgressors’ characteristics such as their social standing. Despite their central role in regulating behavior, many studies have explored moral judgments in response to immoral actions as isolated phenomena, overlooking such contextual influences (Hester & Gray, 2020). This highlights the importance of considering both structural and individual factors when studying moral evaluations.

Consistent with this notion, a growing body of research has recently started to explore how moral judgments are shaped by broader social conditions, such as economic inequality (Kirkland et al., 2024; To et al., 2023). Although such work has offered valuable insights into the role of structural conditions on moral judgments, less attention has been paid to an individual factor that can also influence them: the socioeconomic status (SES) of the transgressor (Polman et al., 2013; Schmidt, 2013; Sharma et al., 2014). To date, few studies have examined the interplay between economic inequality and SES in shaping moral judgments. This gap is particularly important as the two factors are likely to interact, influencing how transgressions are evaluated. To address this, the present study investigates how perceived economic inequality, and the SES of transgressors jointly influence moral judgments. Additionally, it examines the underlying mechanisms that mediate the relationship between economic inequality and moral judgments, such as sense of control and anomie, as well as those that mediate the effect of transgressors’ SES on moral judgments such as sympathy.

### 1.1. Economic Inequality and Moral Judgments

With regard to the effect of economic inequality on moral judgments, authors like To et al. (2023) argue that economic inequality leads to greater leniency. Their findings indicate that the mitigating effect of economic inequality on the severity of moral judgments is due to such inequality reducing sense of control, which increases the likelihood of people thinking that external forces—rather than personal agency—shape one’s life (Kraus et al., 2009; Sirola & Pitesa, 2018). Consequently, in contexts of greater inequality, people may be more inclined to perceive immoral behaviors as being shaped by external constraints rather than deliberate choices, resulting in a greater tolerance for transgressions. Thus, as economic inequality erodes the sense of control, it fosters a social environment in which transgressions are viewed as more acceptable (Casara et al., 2022; Jetten et al., 2021; Malle et al., 2014; Monroe et al., 2017).

Conversely, Kirkland et al. (2024) suggest that, in countries with higher economic inequality, people often make harsher moral judgments toward transgressions. According to their framework, economic inequality leads individuals to view society as fragmented and in a state of anomie. Anomie refers to the perception that society is disintegrating, characterized by a breakdown of social norms and trust in others (Teymoori et al., 2016, 2017). When people experience anomie as a threat, they seek ways to restore order, for instance, by adopting stricter moral judgments (Casara et al., 2022; Kirkland et al., 2024; Sprong et al., 2019). Therefore, economic inequality can foster a heightened sense of anomie, which in turn promotes harsher moral judgments to restore perceived social order.

The evidence reviewed thus far suggests that economic inequality significantly impacts moral judgments. However, inequality has been found to elicit judgments that are both more benevolent and harsher. This variability in findings may arise from the fact that, in studies examining the effects of economic inequality, SES has not been considered as an individual characteristic of the transgressor that may play a crucial role in shaping moral judgments. This gap highlights the necessity of investigating how the SES of the transgressor interacts with economic inequality to shape moral evaluations. In societies marked by economic inequality, it is important to recognize that such systems comprise individuals from varying social strata, whose status may influence judgments of their actions. In fact, economic inequality tends to accentuate the relevance of SES (Jetten et al., 2017; Manstead et al., 2020). Thus, the following question arises: Does economic inequality elicit harsher or more lenient judgments depending on whether the moral transgressor is of high or low status? Indeed, research indicates that the SES of the transgressor influences how their actions are judged (Sharma et al., 2014; Weiner & Laurent, 2021).

### 1.2. Socioeconomic Status and Moral Judgments

The effect of the transgressor’s SES on moral judgments varies, with different patterns emerging for individuals of high and low status. For those of low status, moral judgments often lean toward leniency. Despite a general belief that financial difficulties should not excuse immoral behaviors (Sharma et al., 2014), individuals who are experiencing economic deprivation frequently receive lenient moral judgments (Mullen & Nadler, 2008). Sympathy appears to be a key factor driving such leniency. Previous studies demonstrate that sympathy toward a transgressor plays a significant role in shaping moral evaluations, tending to mitigate blame and increase forgiveness (Koo et al., 2024; Polman et al., 2013). Therefore, since low-SES individuals are more often perceived to be disadvantaged, they are more likely to elicit sympathy, which may, in turn, lead to more lenient moral judgments and greater justification of their actions (Gino & Pierce, 2010; Polman et al., 2013). In contrast, moral evaluations of high-SES individuals are more ambivalent. While their transgressions may, at times, be downplayed in ambiguous situations (Polman et al., 2013), the perceived competence and control of such individuals create higher expectations of exemplary behavior. As a consequence, their moral violations are more likely to be attributed to personal failings, eliciting harsher judgments and punitive responses (Fragale et al., 2009, 2011; Weiner & Laurent, 2021). Moreover, in contexts of greater economic inequality, negative perceptions of wealthy individuals may amplify these harsh judgments further (Schwartz et al., 2021).

Thus, while prior studies have separately demonstrated the relevance of economic inequality and SES on moral judgments, the present study aims to advance this literature by exploring how the two factors interact to influence these judgments. To achieve this, we examine how the two influence moral judgments across three key dimensions: Judgment of the transgression, moral Justification, and the Dehumanization of the transgressor.

### 1.3. Judgment of the Transgression, Moral Justification, and Dehumanization

The first dimension—judgment of the transgression—refers to the evaluation of the severity and immorality of a behavior, alongside the perceived need for punishment. These judgments guide the moral evaluation of the act and the subsequent social consequences. Prior research has shown that both economic inequality and SES shape these judgments, but they have been mostly examined separately, and little is known about how these factors interact, highlighting the need to study how they influence each other (Kirkland et al., 2024; To et al., 2023; Weiner & Laurent, 2021).

Additionally, the negative reactions elicited by immoral behaviors can diminish when reasons for justifying such behaviors are identified (Leidner et al., 2010; Li et al., 2020; Shaw et al., 2003). This process, which is known as moral justification, involves interpreting transgressions as acceptable by identifying arguments to support such actions (Bandura, 1991; Tsang, 2002). In this regard, economic inequality contributes to an environment of social disorder and perceived injustice by highlighting the limited opportunities for upward social mobility, which may facilitate moral justification through situational factors that cause transgressions to appear more justifiable (Davidai, 2018; McCall et al., 2017; Tay et al., 2014). For instance, Koo et al. (2024) demonstrate that when people perceive society as unjust due to its failure to support merit-based upward mobility, they are more likely to justify others’ moral transgressions. Similarly, research on the role of the SES of the transgressor suggests that immoral actions, such as stealing or lying, are more likely to be attributed to contextual factors when they are committed by individuals of lower SES. In contrast, when transgressors are of higher SES, contextual justifications for their actions are more challenging to identify (Weiner & Laurent, 2021). Such findings stress the need to examine how economic inequality and SES jointly influence moral justifications.

Finally, immoral behaviors may lead to the perception of transgressors as less human, a process referred to as dehumanization (Bastian et al., 2013; Khamitov et al., 2016). This process involves perceiving individuals as lacking the traits considered fundamental to human nature (Gray et al., 2007; Haslam, 2006; Leyens et al., 2000). Moreover, the process enables individuals to diminish the moral worth of wrongdoers and justify harsh judgments and punitive actions (Leidner et al., 2013). Research suggests that structural factors, such as economic inequality and SES, influence how dehumanization unfolds. For instance, in contexts characterized by high economic inequality, individuals of lower SES are particularly vulnerable to dehumanization, often in animalistic terms (Sainz et al., 2019, 2024). However, individuals with higher SES are not immune to dehumanization; rather than being likened to animals, they may be dehumanized in mechanistic terms as cold, rigid, or emotionally disconnected (Sainz et al., 2019). Nevertheless, such effects have typically been observed in neutral contexts where judgments are based on group membership or structural conditions rather than on specific behaviors, such as moral transgressions.

While prior research consistently demonstrates that low-SES individuals tend to be dehumanized more frequently—particularly in terms of animalistic dehumanization (Loughnan & Haslam, 2007; Sainz et al., 2019)—these dynamics may shift when individuals are evaluated with consideration of their moral conduct. Moral transgressions activate normative expectations of how people should behave, and these expectations can vary depending on the individual’s social status. Individuals of a high status are typically associated with competence, agency, and social success, and are expected to behave in a responsible, exemplary manner (Fiske et al., 2002; Fragale et al., 2009; Schmidt, 2013). When they violate such expectations, transgressions may be perceived as more serious breaches of moral norms, resulting not only in harsher moral judgments but also a deeper denial of their humanity. This notion aligns with research demonstrating that moral character is central to person perception, and that moral norm violations by individuals who are expected to “know better” evoke particularly intense condemnation (Goodwin et al., 2014; Schmidt, 2013). From this perspective, high-status transgressors may, paradoxically, become more likely to be dehumanized, precisely because their behavior constitutes a stronger violation of moral expectations. Thus, although the roles of inequality and SES have been demonstrated in the dehumanization of groups and individuals, it may be valuable to examine how these socioeconomic factors influence the specific dehumanization of moral transgressors.

**Figure 1:**
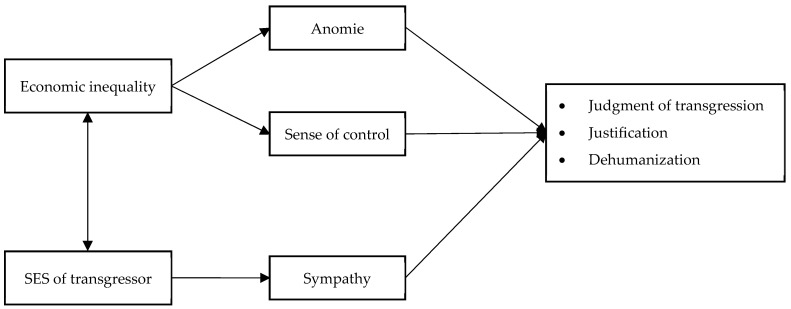
Conceptual framework of studies 1 and 2.

In sum, although prior research has examined the effects of economic inequality and transgressor SES separately, little is known about how the two jointly shape evaluations of moral transgressions and transgressors. The current work proposes an integrative framework in which these factors interact to influence moral judgments across three dimensions: Judgement of the transgression, justification, and dehumanization. Economic inequality is expected to exert its influence via perceptions of anomie and reduced sense of control, whereas transgressor SES is expected to operate through sympathy toward the transgressor. Importantly, the effects of inequality and SES are anticipated to interact, so that the evaluation of a transgression depends on both the societal context and the status of the transgressor. Figure 1 provides a schematic representation of this conceptual framework, which guided the design and hypotheses of Studies 1 and 2.

### 1.4. Overview of the Present Studies

The present research consists of two experimental studies designed to test the effect of economic inequality and SES on moral judgments. For both studies we manipulate the perceived level of economic inequality and the SES of the transgressors. To study underlying mechanisms that explain the effects of each factor on moral evaluations on Study 1 we focus on anomie and sense of control, linked to inequality. In Study 2, we focus on the mediating role of sympathy toward transgressors in shaping the effects of SES on the outcome variables.

Moreover, recent research has highlighted the role of a perceivers’ own SES in shaping social evaluations and dehumanization (Khatry et al., 2021; Piff & Robinson, 2017). While our focus is on contextual inequality and the SES of transgressors, we include perceivers’ SES as a covariate in both studies to account for its potential influence. Data and materials can be found at: https://osf.io/6n94z/?view_only=13a4896a802f45afbcb6e604379b4f74 (accessed 23 September 2025).

## 2. Study 1

Study 1 tests the effects of economic inequality and transgressor’ SES on Judgments of the transgression, Justification, and the Dehumanization of the transgressor. To address these research questions, we first conducted a MANOVA including the three dependent variables, we followed up with univariate ANOVAs to evaluate our specific hypotheses:

- H1: Transgressions committed by high-status individuals will be judged more harshly when committed in unequal societies than when committed in equal societies (H1.1). Conversely, transgressions committed by low-status individuals will be judged less harshly when committed in unequal societies than when committed in equal societies (H1.2).
- H2: Moral transgressions will be more justified when committed in unequal societies than in equal societies (H2.1) or by low-status individuals than by high-status individuals (H2.2).
- H3: High-status transgressors will be more dehumanized in unequal societies than in equal societies (H3.1), while low-status transgressors will be less dehumanized in unequal societies than in equal societies (H3.2).

Furthermore, this study examines the combined mediating roles of anomie and sense of control in these relationships. In addition, we study the mediating role of attitudes toward equal or unequal societies. The materials and results for this last measure can be found in the OSF repository.

### 2.1. Methods

#### 2.1.1. Participants and Design

The study followed a 2 (economic inequality: unequal vs. equal) × 2 (the SES of transgressors: high vs. low SES) between-subjects design. G*Power 3.1 (Faul et al., 2007) indicated that, for a medium effect size (*f* = 0.25) with 95% power (*α* = 0.05), we required 279 participants. We used Prolific.com to recruit 318 participants, of which 29 were excluded for failing attention/comprehension checks or not completing the questionnaire. The final sample comprised 289 participants, all Spanish residents (138 women, 144 men, 7 non-binary) aged between 19 and 50 (*M* = 30.47, *SD* = 8.37). Once participants agreed to partake in the study, they were randomly assigned to one of four experimental conditions.

#### 2.1.2. Materials and Procedure

##### Manipulation of Economic Inequality

To manipulate economic inequality, we followed Tanjitpiyanond et al.’s (2022) method. We presented participants with a list of six fictitious countries with varying levels of inequality. Before evaluating these countries, we explained the functioning of the GINI coefficient to the participants; this was necessary to ensure they understood how the index works, as it would be used to indicate each country’s level of inequality. Depending on the experimental condition, participants were assigned to either the most unequal country on the list (i.e., Nahm) or to the most equal (i.e., Dinh). Participants were provided with a general description of their assigned country (e.g., population, culture, climate), along with a more detailed description of its economy. Thus, for the unequal country, participants saw that the wealthiest 5% gained 150 times more money than the poorest 5%. In contrast, in the equal country, the wealthiest 5% gained only 1.5 times more than the poorest 5%. To provide more information in regard to the level of economic inequality, we established a comparison between the economic inequality of the fictitious country and that of other known countries, such as Iceland, Spain, and South Africa. In addition, throughout the manipulation, we asked participants questions to ensure they understood how the GINI index works. Finally, to confirm whether the manipulation of economic inequality was effective, participants were required to indicate how unequal they considered their assigned country to be on a scale from one (*not unequal*) to nine (*highly unequal*). For more information, please refer to the OSF repository.

##### Manipulation of SES

To manipulate the SES of the transgressors, participants were presented with a fictitious newspaper article detailing an investigation into criminal activity in Nahm (vs. Dinh). The article reported findings from interviews with high-ranking police officials, alongside police and court reports, indicating a pattern of crimes frequently committed by high-SES individuals (vs. low-SES individuals). More specifically, the crimes described included tax fraud, accepting bribes, scamming, making threats, and property theft. The crimes remained identical across all conditions, with only the SES of the perpetrators being altered. These transgressions were selected based on prior literature (i.e., To et al., 2023) and were refined through a pilot study. Our goal was to include a range of behaviors that could plausibly be attributed to individuals with both high- and low-SES. The pilot study revealed that, overall, participants associated most transgressions equally with both groups, except for tax fraud, which was more often linked to high-SES individuals (*M* = 5.45; *SD* = 0.17), and theft, which was more associated with low-SES individuals (*M =* 3.38; *SD* = 0.17; *p* < 0.001). We opted to retain the full set of transgressions to ensure diversity in the scenarios and confirmed that including or excluding these particular items did not substantially alter the pattern of the results.

##### Judgement of the Transgressions

To evaluate participants’ perceptions of transgressions, we asked them to assess each one across various dimensions encompassing moral Judgements (Malle, 2021). Specifically, participants were required to rate the severity, punishability, and immorality of each transgression, with all items ranging from one (*not at all*) to seven (*totally*). For each participant, we then averaged these ratings across all transgressions to create a “Judgment of transgressions” score, with higher scores indicating harsher judgments (α = 0.96).

##### Moral Justification

To determine whether participants considered that different moral Justifications served to exonerate the transgressors and their actions, we employed a number of justifications, which were grounded in prior literature. Specifically, we developed four items representing different forms of justification, based on Kaptein and Van Helvoort’s (2019) classification and Gill and Andreychik’s (2014) Social Explanatory Styles Questionnaire (SESQ) (e.g., “Their context and life experiences have pushed them to do so,” “They are not aware of the real consequences of their actions,” “They do this because everyone does”; α = 0.89). Participants were asked to indicate their level of agreement with each justification for every transgression on a scale from one (*not at all*) to seven (*totally*). The mean of the item scores was calculated to reflect the extent to which participants employed different justifications for transgressions. To ensure that the different items of Moral justification measured a coherent construct, we conducted a principal component analysis. The analysis indicated that all items loaded predominantly on a single factor, with factor loadings ranging from 0.461 to 0.669. The mean of all item scores was calculated to create a composite Moral justification score for each participant.

##### Dehumanization of Transgressors

To measure whether the moral transgressors were dehumanized, we employed an explicit dehumanization measure, following Chen-Xia et al.’s (2022) adaptation of the Ascent of Human Scale (Kteily et al., 2015). We asked participants to answer the following question: “Taking into account what you read previously, if you had to encapsulate your impression of the wealthiest (vs. poorest) people of Nahm (vs. Dinh) at one point on a human-animal scale, where would you place them?” Responses were given on a slider ranging from 0 (“more like an animal”) to 100 (“more like a human”). For ease of interpretation, scores were reverse-coded so that higher values indicated greater dehumanization.

##### Anomie

We measured anomie using the breakdown of social fabric scale developed by Teymoori et al. (2016), as this dimension of anomie is related to the moral domain (Kirkland et al., 2024; Teymoori et al., 2017). Participants were asked to indicate their level of agreement with a series of statements about their assigned country (e.g., “People think that there are no clear moral standards to follow,” “People do not know who they can trust and rely on”; α = 0.92). Responses were given on a scale from one (*not at all*) to seven (*totally*).

##### Sense of Control

To measure sense of control, we employed To et al.’s (2023) adaptation of Kraus et al.’s (2009) sense of control scale. Participants had to indicate, on a scale from one (*not at all*) to seven (*totally*), their agreement with a series of statements about how they would feel if they lived in Nahm or Dinh, depending on their condition (e.g., “I could do just about anything I really set my mind to”; α = 0.89).

##### Sociodemographic Data

We asked participants about their gender, age, and educational attainment, with primary education as the lowest level and college studies the highest. We also asked participants for their political leaning, using a scale from one (*left-wing*) to ten (*right-wing*). Finally, we employed the McArthur Scale of Subjective Social Status (Adler et al., 2000) to measure the subjective social class participants attributed to themselves (ten-point scale from one [*low-SES*] to ten [*high-SES*]).

### 2.2. Results

Before conducting the main analyses, we assessed the effectiveness of the experimental manipulation. To this end, we performed an independent *t*-test to verify whether the participants perceived different levels of economic inequality across conditions. As expected, the results indicated that the participants in the unequal condition perceived significantly higher economic inequality (*M =* 8.59, *SD =* 0.79) compared to those in the equal condition (*M =* 1.55, *SD =* 1.02; *t* (287) = 65.87, *p* < 0.001, *d* = 7.751). As the manipulation was successful, we proceeded with the main analyses.

To test our hypotheses about the three main dependent variables (Judgment of the transgression, Moral Justification, and Dehumanization), we first conducted a MANOVA to assess the impact of economic inequality and SES of the transgressor on the dependent variables. The multivariate test revealed significant main effects of economic inequality (Wilks λ = 0.942, *F*(3,283) = 5.783, *p* = 0.001; η^2^_p_ = 0.058) and SES of the transgressor (Wilks λ = 0.680, *F*(3,283) = 44.374, *p* < 0.001; η^2^_p_ = 0.320). The multivariate interaction was also significant (Wilks λ = 0.930, *F*(3,283) = 7.064, *p* < 0.001; η^2^_p_ = 0.070), indicating a combined effect of inequality and SES on the dependent variables.

Afterwards we performed several 2 (economic inequality: unequal vs. equal) × 2 (the SES of the transgressor: high SES vs. low SES) ANOVAs with each variable. All univariate analyses were interpreted in the context of familywise error across the three correlated dependent variables, using a Bonferroni correction (α = 0.017). Any effects that did not remain significant under this more stringent threshold are noted below. The ANOVA results are summarized in Table 1.

#### 2.2.1. Judgment of the Transgression

The analysis examining whether Judgements of transgressions varied across experimental conditions revealed a significant interaction between economic inequality and the SES of the transgressor (*F*(1,285) = 19.675, *p* < 0.001; η^2^_p_ = 0.065). As we predicted in our first hypothesis, transgressions by high-SES individuals were judged more harshly in the unequal condition (*M* = 6.67, *SD* = 0.40) than the equal condition (*M* = 6.40, *SD* = 0.68; *F*(1,285) = 4.412, *p* = 0.037, η^2^p = 0.015). Furthermore, transgressions by low-SES individuals were judged less harshly in the unequal condition (*M* = 5.30, *SD* = 0.98) than the equal condition (*M* = 5.82, *SD* = 0.81; *F*(1,285) = 17.389, *p* < 0.001, η^2^p = 0.058); this is consistent with H1.2.

#### 2.2.2. Moral Justification

The analysis to examine whether economic inequality or the SES of the transgressor influenced the Justification of moral transgressions revealed a significant main effect of economic inequality (*F*(1,285) = 4.978, *p* = 0.026; η^2^_p_ = 0.017). Concretely, as predicted in H2.1, participants justified transgressions more when they were committed in the unequal condition (*M =* 3.26, *SD* = 1.06) than in the equal condition (*M =* 3.00, *SD* = 0.92). However this effect did not reach significance after applying the Bonferroni correction. Additionally, contrary to our expectations in H2.2, we identified no significant effect of the status of the transgressor. Thus, our second hypothesis was partially supported, as transgressions were more justified in unequal contexts independently of the SES of the transgressor.

#### 2.2.3. Dehumanization of Transgressor

The analysis to verify whether moral transgressors were more dehumanized depending on the level of economic inequality and their SES revealed that, contrary to what H3 proposed, there was a non-significant interaction. Concretely, the results revealed a significant main effect of economic inequality (*F*(1,285) = 10.324, *p* = 0.001; η^2^_p_ = 0.035), as transgressors in the unequal society were more dehumanized *M =* 36.02, *SD* = 29.79) than those in the equal society (*M =* 25.79, *SD* = 24.69). Similarly, we identified a significant main effect of the SES of the transgressor (*F*(1,285) = 26.732, *p* < 0.001; η^2^_p_ = 0.086). More specifically, high-SES transgressors were more dehumanized (*M =* 39.13, *SD* = 29.89) than low-SES transgressors (*M =* 22.95, *SD* = 23.08). Thus, our results indicate that high-SES transgressors and those from unequal societies are more dehumanized.

In addition to the previous analyses, we ran a series of ANCOVAs to check the robustness of our results. In doing so, we employed the same dependent and independent variables of our study but also included the subjective SES of the participants as a covariate. The main results did not change.

#### 2.2.4. Mediation Analyses

We also sought to examine the potential roles of anomie and sense of control in the relationship between economic inequality and our dependent variables. To do so, we employed Hayes’s (2018) PROCESS macro (Model 4, 95% CI, 10,000 bootstrap samples). In simple mediation models, anomie significantly mediated the effect of inequality on Justification (*B* = 0.49, *SE* = 0.12, 95% CI [0.251, 0.729]) and on Dehumanization (*B* = 11.41, *SE* = 3.52, 95% CI [4.685, 18.436]). Sense of control did not mediate the effect of inequality on justification (*B* = 0.12, *SE* = 0.065, 95% CI [−0.001, 0.258]), nor in dehumanization (*B* = 2.59, *SE* = 1.79, 95% CI [−0.762, 6.309]). For judgment of transgression, mediation was not significant. Full details are available in the OSF repository.

**Figure 2:**
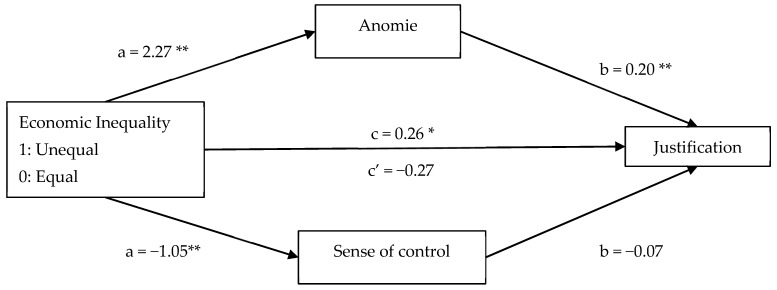
The mediational roles of anomie and sense of control on the relationship between economic inequality and Justification. *Note.* Unstandardized regression coefficients are reported with 95% CI. * *p* < 0.05, ** *p* < 0.001.

**Figure 3:**
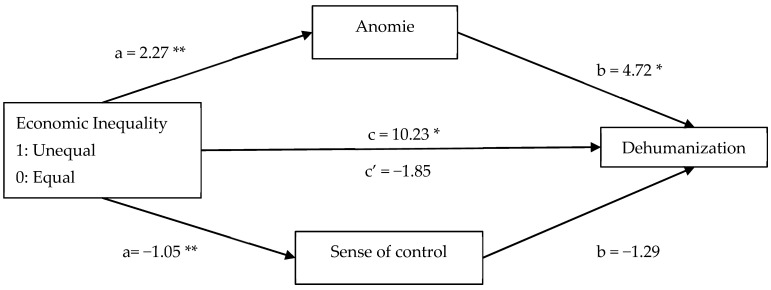
The mediational roles of anomie and sense of control on the relationship between economic inequality and Dehumanization. *Note.* Unstandardized regression coefficients are reported with 95% CI. * *p* < 0.05, ** *p* < 0.001.

Building on these results, we conducted parallel mediation analyses including both mediators simultaneously. The analysis highlighted a significant total effect of economic inequality on Justification (*B* = 0.26, *SE* = 0.12, *p* = 0.029, 95% CI [0.026, 0.489]). When the mediators were included, the direct effect became non-significant (*B* = −0.27, *SE* = 0.17, *p* = 0.114, 95% CI [−0.604, 0.065]), suggesting that the relationship between inequality and Justification was explained through the indirect pathways. As shown in Figure 2, only the conditional indirect effect of anomie was significant (*B* = 0.46, *SE* = 0.13, 95% CI [0.204, 0.716]), whereas the conditional indirect effect of sense of control was not (*B* = 0.07, *SE* = 0.07, 95% CI [−0.058, 0.205]). These indirect effects are conditional on the inclusion of both mediators in the model and may be influenced by potential suppression. For Dehumanization (see Figure 3), the parallel mediation also showed a significant conditional indirect effect of anomie (*B* = 10.72, *SE* = 3.68, 95% CI [3.486, 18.137]), and a non-significant conditional effect of sense of control (*B* = 1.35, *SE* = 1.79, 95% CI [−2.094, 4.938]) indicating that anomie mediates the effect of inequality on Dehumanization even when controlling for sense of control. These indirect effects are conditional on the inclusion of both mediators and may be influenced by potential suppression.

Overall, the findings suggest that the tendency to justify transgressions and dehumanize transgressors in unequal contexts operates primarily through perceived anomie.

### 2.3. Discussion

In conclusion, this study demonstrates that both economic inequality and the SES of moral transgressors significantly influence judgment of transgressions and those who commit them. Specifically, it identifies that, as suggested by previous studies, economic inequality leads to transgressions being judged either more harshly or more leniently (Kirkland et al., 2024; To et al., 2023). As hypothesized, the difference in the direction of these judgments is attributed to the transgressor’s status. In this sense, transgressions committed by high-SES perpetrators are judged more harshly in economically unequal contexts than in equal contexts. Meanwhile, for low-SES perpetrators, transgressions are judged less harshly when committed in economically unequal contexts than in equal contexts.

Furthermore, economic inequality influences the Justification of transgressions; individuals exhibit a greater tendency to justify such misconduct when it occurs in unequal contexts. This effect of economic inequality on Moral Justification is mediated primarily by individuals’ perceptions of greater levels of anomie in unequal societies. The increased role of anomie in Moral Justification may arise from the attribution of such transgressions to the social unrest and disorder that can characterize economically unequal and anomic societies (Teymoori et al., 2017).

Finally, moral transgressors are more likely to be dehumanized when they are of a higher SES and when their transgressions occur within an unequal context, although these factors operate independently. Interestingly, existing research on dehumanization and socioeconomic inequalities has consistently found that low-SES individuals are more prone to dehumanization (Sainz et al., 2019, 2024). However, this study demonstrates that, when considering the impact of SES on the dehumanization of moral transgressors, high-SES individuals are subjected to a greater loss of human characteristics compared to low-SES individuals. Furthermore, perceived anomie mediates the effect of inequality on the dehumanization, suggesting that in unequal contexts, individuals dehumanize more the transgressors due to perceiving more anomie.

In summary, our findings indicate that both economic inequality and the SES of moral transgressors influence judgments regarding the transgressions and the perpetrators. Moreover, our results demonstrate that anomie mediates the effect of economic inequality on Moral Justifications and Dehumanization. In contexts perceived as socially disordered, individuals may simultaneously justify deviant behaviors and dehumanize the transgressors more. These findings highlight the dual role of anomie in shaping the relationship between economic inequality and moral judgments. However, we did not find evidence that a reduced sense of personal control mediated the effect of inequality on moral judgments, even though past work (To et al., 2023) had suggested this pathway. It appears anomie was more consequential than personal control perceptions in this unequal context. In our second study, we sought to replicate these findings and further investigate the mediating mechanisms that potentially explain the effect of transgressors’ SES Judgments of their transgression, Moral Justification, and Dehumanization.

## 3. Study 2

Building on the findings of Study 1, Study 2 aims to replicate and extend the previous results. As the first study did not address the potential factors that influence the tendency to be more punitive towards high-status transgressors, Study 2 examines how attitudes toward high- and low-SES individuals can shape these moral evaluations (Colbow et al., 2016; Koo et al., 2024; Polman et al., 2013). One key aspect of this investigation is the role of sympathy toward the transgressor. Prior studies have indicated that the level of sympathy toward those who transgress moral norms can significantly influence their level of blame, the likelihood of moral license being granted, or whether the actions of the transgressor are justified (Eaton & Struthers, 2006; Koo et al., 2024; Polman et al., 2013). Moreover, feelings of sympathy are linked to SES, with people tending to feel more sympathy toward those experiencing economic hardship, which can, in turn, lead to more lenient Judgements of moral transgressions of such individuals (Polman et al., 2013). Based on this, for Study 2 we maintain the same hypothesis of the first study and also propose that sympathy toward the transgressor will mediate the relationship between SES, Judgments of the transgression, Justification, and Dehumanization (H4). We note that this hypothesis was not preregistered and is presented here as a transparent extension of our preregistered plan.

Furthermore, due to the need to isolate the effect of sympathy toward the target while also accounting for potential real-world biases or classist attitudes toward individuals from different socioeconomic backgrounds, classism is included as a control variable in the mediation analyses. This allows us to examine whether classist attitudes influence the effects of SES on moral judgments and Dehumanization.

All analyses followed the preregistration plan (OSF link). The preregistration specified ANOVAs as the primary tests for our dependent variables. For completeness, we also report MANOVA as a robustness check; this was not preregistered but is consistent with the logic of testing correlated outcomes. Preregistration can be found at: https://osf.io/ksvaq?view_only=13a4896a802f45afbcb6e604379b4f74 (created on 17 July 2024).

### 3.1. Methods

#### 3.1.1. Participants and Design

The study followed a 2 (economic inequality: unequal vs. equal) × 2 (SES of transgressors: high SES vs. low SES) between-subjects design. G*Power (Faul et al., 2007) indicated that, for a medium effect size (f = 0.25) with 95% power (α = 0.05), we needed 279 participants. To replicate Study 1 and to conduct new mediation analyses regarding sympathy, we recruited 567 participants via Netquest. From this number, we excluded 166 participants who did not complete the questionnaire or who failed the control questions. The final sample comprised 401 participants (206 women, 195 men; *M*_age_ = 43.80, *SD*_age_ = 12.78). Once participants agreed to partake in the study, they were randomly assigned to one of the experimental conditions.

#### 3.1.2. Materials and Procedure

As the primary objective of Study 2 was to test the effect of sympathy toward the transgressor on the relationship between the SES of the transgressor and moral judgment, we introduced two new measures:

##### Sympathy Toward the Transgressor

To assess whether participants feel more sympathy toward high- or low-SES transgressors, we employed Polman et al.’s (2013) measure of sympathy Participants in the high-SES transgressor condition rated the extent to which they felt sympathy toward the wealthy individuals who committed the transgressions, whereas participants in the low-SES transgressor condition rated sympathy toward the poor individuals who committed the transgressions. Participants gave their answers on a scale from one (*not at all*) to seven (*totally*).

##### Upward and Downward Classism

We employed classism as a control variable and adapted Colbow et al.’s (2016) Classism Attitudinal Profile (CAP). This scale comprises two differentiated types of classism, one directed to the lower class (e.g., “People who are poor let their kids run around without supervision”; α = 0.77) and the other directed to the upper class (e.g., “Rich people’s kids are troublemakers”; α = 0.81).

To manipulate economic inequality and SES, we employed the same materials and procedure used in Study 1. The measures of moral judgments also remained the same as those used in Study 1: Judgment of transgressions (α = 0.96); Moral Justification (α = 0.94), and Dehumanization of transgressors. Finally, we also included the measure of anomie (α = 0.87) to check whether the results of Study 1 were replicated.

### 3.2. Results

Prior to the analyses of the main data, we verified the success of the experimental manipulation. To do so, we carried out the same *t*-test performed in Study 1, as expected participants perceived different levels of inequality between the conditions. More specifically, participants perceived higher economic inequality in the unequal condition (*M =* 7.38, *SD =* 2.37) than in the equal condition (*M =* 2.96, *SD =* 2.58; *t* (399) = −17.949, *p* < 0.001, *d* = −1.79). Given that this preliminary test ensured the effectiveness of our manipulation, we proceeded with the main analyses of the study.

To test the hypotheses about our three main dependent variables, we followed the same analysis plan as in Study 1. The multivariate test revealed significant main effects of economic inequality (Wilks λ = 0.973, *F*(3,395) = 3.657, *p* = 0.013; η^2^_p_ = 0.027) and SES of the transgressor (Wilks λ = 0.840, *F*(3,395) = 25.088 *p* < 0.001; η^2^_p_ = 0.160) on the combined dependent variables. However, the interaction between both variables was non-significant (Wilks λ = 0.996, *F*(3,395) = 0.559, *p* = 0.642; η^2^_p_ = 0.004).

Subsequently, we conducted separate 2 (economic inequality: unequal vs. equal) × 2 (the SES of the transgressor: high SES vs. low SES) ANOVAs for each dependent variable. All univariate analyses were interpreted in the context of familywise error across the three correlated dependent variables, using a Bonferroni correction (α = 0.017). The ANOVA results are summarized in Table 2.

#### 3.2.1. Judgment of the Transgression

The analysis to verify whether there were distinctions in the judgment of transgressions depending on the experimental conditions showed a significant main effect of the status of transgressor (*F*(1,397) = 54.124, *p* < 0.001; η^2^_p_ = 0.120). Concretely, transgressions by high-SES individuals were judged more harshly (*M =* 6.43, *SD* = 0.85) than those by low-SES individuals (*M =* 5.71, *SD* = 1.06). That is, transgressions committed by high-SES individuals are perceived as worse than those committed by low-SES individuals, independently of the level of economic inequality within the society. However, contrary to our hypothesis and the findings of Study 1, there was no interaction between economic inequality and the SES of the transgressor.

#### 3.2.2. Moral Justification

The ANOVA to check whether economic inequality and the SES of the transgressor influenced the Justification of moral transgressions demonstrated a significant main effect of economic inequality (*F*(1,397) = 7.096, *p* = 0.008; η^2^p = 0.018). Concretely, as expected in H2.1 and replicating the results of Study 1, transgressions were more justified when committed in unequal societies (*M =* 3.79, *SD* = 1.36) than in equal societies (*M =* 3.42, *SD* = 1.41). In addition, in Study 2, we determined a main effect of status (*F*(1,397) = 6.826, *p* = 0.009; η^2^p = 0.017), aligned with H2.2. Participants justified the transgressions of low-SES individuals (*M =* 3.79, *SD* = 1.20) more than those of high-SES individuals (*M =* 3.42, *SD* = 1.54).

**Figure 4:**
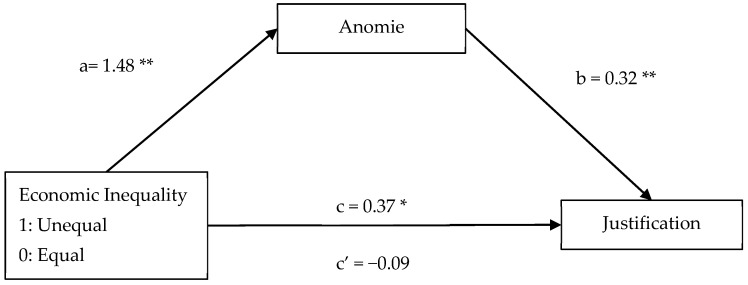
The mediational role of anomie on the relationship between economic inequality and Moral Justification of the transgressions. *Note.* Unstandardized regression coefficients are reported with 95% CI. * *p* < 0.05, ** *p* < 0.001.

Furthermore, to test whether anomie mediated the relationship between economic inequality and Justification, we employed Hayes’s (2018) SPSS macro PROCESS version 4 (Model 4, 95% CI, 10,000 bootstrap samples) with anomie as a mediator. As seen in Figure 2, there was a significant total effect of economic inequality on the Justification of transgressions (*B* = 0.37, *SE* = 0.14, *p* = 0.008), which became non-significant when the mediator was introduced (*B* = −0.09, *SE* = 0.15, *p* = 0.515). In addition, the indirect effect of anomie was significant (*B* = 0.36, *SE* = 0.07, 95% CI [0.218, 0.713]). Therefore, as in Study 1, the results demonstrate that people tend to justify more moral transgressions committed in unequal contexts via perceiving more anomie (Figure 4).

#### 3.2.3. Dehumanization of Transgressor

The analysis testing whether moral transgressors were more dehumanized depending on the level of economic inequality and their SES demonstrated a significant main effect of status (*F*(1,397) = 23.99, *p* < 0.001; η^2^_p_ = 0.057). Replicating the findings of Study 1, high-SES transgressors were more dehumanized (*M =* 53.14, *SD* = 32.49) than low-SES transgressors (*M =* 38.26, *SD* = 28.44). However, contrary to our expectations, there was no effect of economic inequality; that is, high-SES transgressors were always more dehumanized independently of the level of economic inequality. Additionally, mediation analyses showed that perceived anomie did not significantly mediate the effect of economic inequality on Dehumanization (B = 0.99, *SE* = 1.89, 95% CI [−2.622, 4.784]) (see OSF repository).

As in study 1, we ran a series of ANCOVAs, employing the same dependent and independent variables, while also including the subjective SES of the participants and upwards and downwards classism as covariates. The main results were not influenced by these variables.

#### 3.2.4. Sympathy Toward the Transgressor

Since one of the main objectives of Study 2 was to understand the role of sympathy in the relationship between the transgressor’s status and the evaluation of their immoral behavior, we conducted a series of mediation analyses with the SPSS macro PROCESS version 4 (Model 4, 95% CI, 10,000 bootstrap samples), with sympathy toward the transgressor serving as the main mediator between SES and our three dependent variables. We also controlled for the upward and downward classism of the sample. Including classism as a covariate did not affect the results, and sympathy remained a significant mediator. Reported below are all the mediation analyses, direct, total, and indirect effects for all mediations are summarized in Table 3. Figures for all analyses can be found in the OSF repository.

Firstly, the mediation analyses demonstrated a significant effect of the SES of the transgressor on sympathy (*B* = −1.49, *SE* = 0.16, *p* < 0.001, 95% CI [−1.797, −1.187]). Focusing on the judgment of the transgression, the analysis of indirect effects revealed that sympathy was a significant mediator between the transgressor’s SES and how they were judged (*B* = 0.20, *SE* = 0.05, 95% CI [0.109, 0.318]). This implies that transgressions committed by high-SES individuals are judged more harshly via having less sympathy toward them.

Regarding Moral Justification, the analysis of the indirect effects showed that sympathy was a significant mediator (*B* = −0.29, *SE* = 0.07, 95% CI [−0.452, −0.149]), which supported our hypothesis. Therefore, the results demonstrated that people tend to justify less moral transgressions committed by high-SES transgressors via having less sympathy toward them.

Finally, the mediation analysis for Dehumanization also demonstrated a significant effect of sympathy on the relationship between the SES of the transgressor and Dehumanization (*B* = 12.23, *SE* = 1.88, 95% CI [8.755, 16.116]). Thus, people tend to dehumanize high-SES transgressors more via having less sympathy toward them.

### 3.3. Discussion

In conclusion, Study 2 provides further evidence that both economic inequality and the SES of moral transgressors shape responses to immoral actions. While we did not find the predicted interaction between these two factors, the findings highlight the distinct main effects contributing to a more nuanced understanding of how economic inequality and SES influence moral judgments. More specifically, we identified that transgressions by high-SES individuals were judged more harshly than transgressions committed by low-SES individuals independently of the economic inequality level. Similarly, we found that high-SES transgressors were more dehumanized than low-SES transgressors, irrespective of the level of economic inequality, and that anomie did not influence the Dehumanization of these transgressors. Furthermore, as we proposed, the tendency to judge transgressions more harshly and dehumanize high-SES transgressors more is primarily attributed to having less sympathy toward them.

Interestingly, with this study, we determined that both economic inequality and SES affect Justification. Firstly, supporting the previous study, transgressions in unequal societies were more justified via a higher perception of anomie. Additionally, the study also indicates that people’s tendency to justify more the transgressions committed by low-SES individuals, rather than the transgressions of high-SES individuals, can be explained by feeling more sympathy toward the low-SES group.

## 4. General Discussion

This study examined how economic inequality and SES jointly influence moral judgments. To date, the effect of these factors on moral judgments has been studied separately, leading to different patterns in terms of their impacts on the judgment of immoral actions. Our approach sought to address this gap by offering a broader framework for understanding how these two elements interact. Although our findings did not fully support the predicted interaction effects across the studies, they nevertheless offer insights into the distinct roles of inequality and status in shaping moral evaluations and the psychological mechanisms through which these effects occur.

In our first hypothesis for both studies, we expected that those transgressions committed by high-SES individuals would be judged more harshly in contexts of high inequality compared to those of low inequality. Conversely, we expected the opposite pattern with low-SES transgressors, with their transgressions judged less harshly in unequal contexts. Overall, our findings indicate that the SES of the transgressor consistently influences the judgment of the transgressions, while the role of economic inequality varies across studies. On the one hand, the results of Study 1 demonstrate that economic inequality influences the judgment of the transgression differently depending on the SES of the transgressor. Furthermore, these findings are consistent with prior research indicating that higher economic inequality and SES are associated with a greater tendency to judge immoral behaviors more harshly (Kirkland et al., 2024; Polman et al., 2013). On the other hand, in Study 2, moral judgments of the transgression were only significantly affected by the SES of the transgressor. Concretely, and consistent with the findings of Fragale et al. (2009, 2011) and Weiner and Laurent (2021), transgressions committed by high-SES individuals were judged more harshly compared to those committed by low-SES individuals. In addition, Study 2 revealed that transgressions by high-SES individuals were judged more harshly due to a diminished sympathy toward them. Thus, as Polman et al. (2013) proposed, we found that lower sympathy toward high-SES transgressors explains why people tend to be more punitive when judging their misdeeds. Additionally, the fact that the interaction between inequality and SES observed in Study 1 did not replicate in Study 2 may reflect differences in participant age or sample composition, suggesting that sensitivity to inequality could vary across populations. Taken together, the findings from Studies 1 and 2 indicate that, while economic inequality shapes moral judgments, the SES of the transgressor exerts a more substantial and consistent influence on the evaluation of transgressions, sometimes independent of the level of economic inequality in the broader context.

Regarding our second hypothesis, we expected moral transgressions to be more justified in unequal contexts than in equal contexts, as well as when committed by low-SES individuals than by high-SES individuals. As expected, in both studies, we found that moral transgressions were more justified when committed in unequal societies than in equal ones. Interestingly, we identified anomie as the mechanism that explains this effect of economic inequality on Moral justification. More specifically, greater inequality increased perceptions of anomie in the society, and, in turn, increased Moral justification. This finding offers a relevant contrast with the work of Kirkland et al. (2024), in which anomie is described as a mechanism that leads to harsher moral judgments. Our results, in contrast, reveal that perceptions of anomie in unequal societies foster greater understanding of immoral behaviors. The fact that anomie increases Moral justification in contexts of high inequality may be explained because, in such situations, anomie generates a perception of social disintegration and the breakdown of norms (Teymoori et al., 2017; To et al., 2023). Within this context, individuals may attribute others’ transgressions to this state of social disorder, making it easier to find justifications for why such transgressions occur. This mechanism indicates that the perception of a chaotic social environment—driven by high economic inequality—may be significant in the interpretation and justification of immoral behaviors. Moreover, Study 2 also demonstrated that moral transgressions were more justified when committed by low-SES individuals than by high-SES individuals. This tendency to justify the transgressions of low-SES individuals aligns with the proposals of Weiner and Laurent (2021) and Fragale et al. (2009, 2011). Our results determined that sympathy toward low-SES individuals serves as a key mechanism underlying this increased justification. Consistent with the findings of Polman et al. (2013), people are more likely to feel sympathy for those in economically precarious situations, which, as observed in our study, may contribute to greater justification of transgressions.

Finally, our third hypothesis proposed that high-SES transgressors would be more dehumanized in contexts of high inequality, whilst low-SES transgressors would be less dehumanized in such contexts. However, across both studies, we identified a consistent pattern: high-SES transgressors were more likely to be dehumanized, regardless of context. This finding highlights an intriguing phenomenon. In general, prior studies have revealed a greater tendency to dehumanize individuals of low SES, often perceiving them to be less evolved and associating them with animalistic traits—an effect that is intensified in contexts of high inequality (Sainz et al., 2019, 2024). Conversely, our studies indicate that, in the case of moral transgressors, high-SES individuals are stripped of more human characteristics compared to their low-SES counterparts. Notably, the lack of a consistent effect of economic inequality on dehumanization may explain why perceived anomie did not emerge as a stable predictor in this context. The fact that the high-SES transgressors are more dehumanized is in line with previous studies indicating that immoral behaviors by wealthy individuals are typically judged more harshly (Fragale et al., 2009, 2011; Polman et al., 2013; Schmidt, 2013; Schwartz et al., 2021; Weiner & Laurent, 2021). Furthermore, our findings expand the existing body of work by demonstrating that such negative evaluations are accompanied by greater dehumanization. In addition, we corroborate that sympathy acts as a key explanatory mechanism for this phenomenon. People typically feel less sympathy for individuals of high SES, in turn facilitating their dehumanization when they commit moral transgressions.

In summary, this research demonstrates that both economic inequality and the SES of a moral transgressor influence how immoral behaviors—and those who commit them—are evaluated. Specifically, economic inequality provides a broader context facilitating the justification of transgressions, particularly through increased perceptions of anomie. Furthermore, across both studies, we observed a consistent effect of the SES of the transgressor: high-SES individuals were more dehumanized, and their transgressions were judged more harshly than those committed by low-SES individuals due to reduced sympathy towards these high-SES individuals

### Limitations and Future Studies

Our studies undoubtedly have some limitations. Firstly, while we determined the sample sizes a priori using effect size calculations, the complexity of the designs—including mediating variables—suggests that future research might benefit from larger samples to increase statistical power and enhance the reliability of the findings. Additionally, the studies relied on hypothetical, highly controlled scenarios to manipulate both economic inequality and the SES of the transgressor. Although such experimental designs provide higher internal validity and allow causal inference, their ecological validity is inherently limited. The use of fictitious societies or imagined contexts, while common in this type of research, may not fully capture the complexity of participants’ real-life experiences with inequality and social class. Future research may benefit from incorporating more ecologically grounded methods to complement the current findings.

Secondly, the manipulation we employed focused on immoral behaviors related to economic crimes. While the same behaviors were used for both high- and low-SES individuals, different patterns may emerge with other types of offenses. Prior research has demonstrated that dehumanization can vary depending on the nature of the crime (Bastian et al., 2013). Additionally, both studies were conducted with Spanish participants in an online setting, and cultural or regional factors might influence how inequality and status are perceived. Future research could explore whether the pattern we observed—greater punitiveness toward high-SES individuals, particularly in contexts of high inequality—also applies to other forms of immoral behavior and in samples from different cultural or national backgrounds. This would help establish the generalizability of our findings and determine whether SES-based moral judgments operate similarly across diverse domains of wrongdoing and populations.

Thirdly, the absence of standardized scales to measure moral justification led us to develop a specific scale based on existing theoretical frameworks (Gill & Andreychik, 2014; Kaptein & Van Helvoort, 2019). Although this approach is common in studies on moral rationalization, it may have introduced methodological challenges. Moreover, we primarily focused on whether immoral actions are justified to a greater or lesser extent depending on inequality and SES. Future research might explore whether people are more inclined to accept certain types of justifications over others, depending on the context of the immorality and transgressors’ characteristics. Furthermore, while our findings demonstrated that sympathy acts as a key mechanism explaining why individuals are more lenient toward low-SES transgressors, it would be valuable to examine whether other factors—including beliefs about whether wealth or poverty is deserved—affect evaluations of immoral behaviors.

Finally, we consistently identified a pattern of greater dehumanization toward high-SES individuals who act immorally. However, previous research on dehumanization and socioeconomic status suggests that the underlying mechanisms differ depending on individuals’ SES: those with higher SES tend to be mechanized, while those with lower SES are more often animalized (Loughnan & Haslam, 2007; Sainz et al., 2019). Future research should incorporate dehumanization measures that capture such distinctions more precisely and investigate whether, as observed in our studies, high-SES individuals are also more animalized when engaging in moral transgressions.

## 5. Implications and Conclusions

Moral judgments are influenced not only by the nature of the transgression itself, but also by the context in which it occurs and the characteristics of the transgressor. The growing economic inequality of recent years has generated considerable interest in understanding how socioeconomic factors shape these judgments. However, much of the prior research has examined the effects of contextual factors (e.g., inequality) and individual characteristics (e.g., the status of the transgressor) in isolation, leaving a gap in understanding their combined influence. This study addresses this gap and highlights how the different dimensions of moral evaluation (e.g., severity, justification, and dehumanization) are differentially shaped by economic inequality and the SES of the transgressor.

While our main aim was to explore how the two factors interact, our findings indicate that economic inequality appears to primarily influence the justification of immoral behaviors, whereas the judgment of the transgression and the dehumanization of the transgressor are more strongly shaped by the transgressor’s SES. This suggests that the two socioeconomic factors do not operate uniformly across all dimensions of moral evaluation but exert distinct influences depending on the psychological process involved.

Another contribution of this study lies in the consistent finding that high-SES transgressors were more dehumanized than their low-SES counterparts, regardless of the level of economic inequality. This contrasts with much of the existing literature, which has typically documented stronger dehumanization of low-SES individuals, particularly in non-moral contexts (e.g., Loughnan & Haslam, 2007; Sainz et al., 2019). Our findings suggest that when moral transgressions are taken into account, the patterns of dehumanization change: high-SES individuals who commit immoral acts are attributed fewer human qualities than low-SES individuals. This may be because the actions of high-SES transgressors are perceived as particularly unjustifiable or disappointing due to social expectations tied to their position (Schmidt, 2013; Weiner & Laurent, 2021). In this sense, the moral context appears to shift the usual focus of dehumanization from the disadvantaged to the privileged when moral norms are breached. Such findings expand current knowledge by demonstrating that dehumanization is not inherently tied to social disadvantage and can also emerge as a form of moral punishment directed at those perceived to be in a position of greater responsibility or moral accountability.

In this sense, this study provides a comprehensive understanding of how economic inequalities between the most privileged and the most disadvantaged influence moral judgments. These findings demonstrate that economic inequality and SES affect moral evaluations, leading to reduced moral justification and increased dehumanization when high-SES individuals violate social norms.

## Figures and Tables

**Table 1:** Effect of economic inequality and SES on Judgment of the transgression, Justification and Dehumanization for study 1 (Hypotheses 1–3).

		Unequal Condition	Equal Condition	ANOVA		
		M(SD)	M(SD)	Effect	F	η^2^_p_
Judgment of the transgression	High-SES	6.66(0.40)	6.40(0.68)	SES	122.47 **	0.301
	Low-SES	5.30(0.98)	5.82(0.81)	Inequality	2.16	0.008
				SES × Inequality	19.67 **	0.065
Justification	High-SES	3.09(1.24)	2.99(1.08)	SES	2.426	0.008
	Low-SES	3.44(0.81)	3.01(0.74)	Inequality	4.98 *	0.017
				SES × Inequality	1.993	0.007
Dehumanization	High-SES	43.59(32.19)	34.21(26.49)	SES	10.324 **	0.035
	Low-SES	28.15(24.93)	17.60(19.77)	Inequality	26.73 **	0.086
				SES × Inequality	0.036	0.000

*Note*. * *p* < 0.05, ** *p* < 0.001.

**Table 2:** Effect of economic inequality and SES on Judgment of the transgression, Justification and Dehumanization for study 2 (Hypotheses 1–3).

		Unequal Condition	Equal Condition	ANOVA		
		M(SD)	M(SD)	Effect	F	η^2^_p_
Judgment of the transgression	High-SES	6.38(0.93)	6.47(0.78)	SES	54.124 **	0.120
	Low-SES	5.66(1.10)	5.76(1.02)	Inequality	0.941	0.002
				SES × Inequality	0.009	0.000
Justification	High-SES	3.56(1.50)	3.29(1.57)	SES	6.82 *	0.017
	Low-SES	4.02(1.15)	3.55(1.21)	Inequality	7.09 *	0.018
				SES × Inequality	0.509	0.001
Dehumanization	High-SES	57.63(32.49)	48.66(30.69)	SES	23.99 **	0.057
	Low-SES	39.44(28.32)	37.02(28.66)	Inequality	3.50	0.009
				SES x Inequality	1.16	0.003

*Note*. * *p* < 0.05, ** *p* < 0.001.

**Table 3:** Total, direct, and indirect effects (via sympathy) of the transgressor’s SES on judgment of the transgression, Justification, and Dehumanization (Hypothesis 4).

	Total Effect		Direct Effect		Indirect Effect	
	Coeff (SE)	95% CI	Coeff (SE)	95% CI	Coeff (SE)	95% CI
Judgment of transgression	0.65(0.09)	[0.467, 0.833]	0.45(0.10)	[0.248, 0.644]	0.20(0.05)	[0.109, 0.318]
Justification	−0.34, (0.13)	[−0.603, −0.084]	−0.05, (0.14)	[−0.335, 0.228]	−0.29(0.07)	[−0.452, −0.149]
Dehumanization	14.52, (3.07)	[8.482, 20.561]	2.29, (3.11)	[−3.826, 8.401]	12.23(1.88)	[8.755, 16.116]

*Note*. Unstandardized regression coefficients are reported. The mediation model includes sympathy as the sole mediator.

## Data Availability

The materials and original data presented in the study are openly available in osf at https://osf.io/6n94z/?view_only=13a4896a802f45afbcb6e604379b4f74 (accessed 23 September 2025).
